# Long-Term Outcome of Xenon-Arc Photocoagulation for Retinopathy of Prematurity in the 1970s in Japan: Eleven Patients With 32- to 49-Year Follow-Up

**DOI:** 10.7759/cureus.97797

**Published:** 2025-11-25

**Authors:** Toshihiko Matsuo, Nobuhiko Matsuo

**Affiliations:** 1 Healthcare Science, Graduate School of Interdisciplinary Science and Engineering in Health Systems, Okayama University, Okayama City, JPN; 2 Ophthalmology, Okayama University Hospital, Okayama City, JPN; 3 Ophthalmology, Okayama University Medical School, Okayama City, JPN

**Keywords:** 1970s, cataract, cryocautery, japan, late complications, neonatology, retinal detachment, retinopathy of prematurity, vitreous hemorrhage, xenon-arc photocoagulation

## Abstract

Objectives: Photocoagulation or cryocautery, or their combinations, are the standard of care for retinopathy of prematurity at the recommended timing, which is based on the International Classification of Retinopathy of Prematurity. In Japan, the effectiveness of xenon-arc photocoagulation and cryocautery in retinopathy of prematurity was reported on an empirical basis first in 1968, and became the standard of care in retinopathy of prematurity in the 1970s, 10 years earlier compared with the other countries. In this study, we reported the up to 49 years visual outcome of 11 patients with retinopathy of prematurity who underwent xenon-arc photocoagulation and cryocautery in the 1970s.

Methods: A retrospective review was made on the medical records of 11 consecutive patients who underwent xenon-arc photocoagulation for retinopathy of prematurity in the years 1974 to 1980, and were followed up until the period from 2009 to 2025. The birthweight ranged from 865 g to 2300 g at a median of 1350 g, and the gestational age at birth ranged from 27 weeks to 36 weeks at a median of 30 weeks. The corrected gestational age at the time of photocoagulation ranged from 32 weeks to 53 weeks, with a median of 37 weeks. Oxygen was given to all 11 patients, except for one who was born in the earliest year 1974. The retinopathy of prematurity was at stage 3 in both eyes of seven patients, with plus disease signs in four patients, at stage 2 with and without plus disease in two patients, at stage 2 and stage 3 in each eye of one patient, and at stage 1 with plus disease in both eyes of one patient. The entire 360-degree photocoagulation was given in seven patients, while partial photocoagulation was applied in four patients. Additional cryocautery was applied in six patients.

Results: The age at the last visit ranged from 32 to 49 years with a median of 46 years. At the last visit, seven patients showed the best-corrected visual acuity in decimals of 0.8 or better in both eyes. One dizygotic twin showed no light perception in the phthisic right eye and 0.1 in the left eye with macular degeneration and nystagmus after he underwent cataract surgery at the age of 34 years. The other twin had the best-corrected visual acuity of 0.5 in the right eye and 0.02 in the left eye due to macular degeneration after he underwent cataract surgeries in both eyes at the age of 36 years. Two patients developed rhegmatogenous retinal detachment in one eye at the age of 44 and 41 years, respectively, and underwent vitrectomy with silicone oil tamponade, resulting in visual acuity of 0.1 and 0.3, respectively. Two patients experienced vitreous hemorrhage in one eye, which was absorbed spontaneously at the ages of 37 years and 42 years, respectively. One patient underwent partial scleral buckling for localized rhegmatogenous retinal detachment. No patient used intraocular pressure-lowering eyedrops.

Conclusion: Most patients with xenon-arc photocoagulation for retinopathy of prematurity in the 1970s maintained standard levels of visual acuity up to 49 years in the follow-up. Cataract, retinal detachment, and vitreous hemorrhage were noted as late complications and were coped with on an individual basis. The conclusion would have a meaning, even though not novel, that the patients with retinopathy of prematurity would have benefited from the xenon-arc photocoagulation and cryocautery.

## Introduction

In the process of embryonic development, the retinal blood vessels start growing from the optic disc at the gestational age of 14 to 20 weeks and reach the peripheral retina on the temporal side at the gestational age of 36 weeks [1,2]. Abnormal vascular growth, such as abnormal vascular branching and growth into the vitreous, may occur in the retina of babies with premature birth in response to environmental oxygen or oxygen therapy, which is higher in concentration, compared with the intrauterine condition. Major risk factors for retinopathy of prematurity are premature birth and low birth weight. In the standard of care, babies with premature birth at the gestational age of 34 weeks or younger and with a birthweight of 1800 g or smaller are recommended to be monitored by ophthalmologists to check possible development of retinopathy of prematurity. Premature babies, especially born before the gestational age of 28 weeks and with a birthweight of less than 1500 g, are monitored carefully for retinopathy of prematurity [1]. In the modern era, the excess administration of oxygen can be avoided by percutaneous oxygen monitoring.

Retinopathy of prematurity progresses from stage 1 to stage 5: demarcation line formation between the vascularized retina and avascular retina at stage 1, thickening of the demarcation line by intraretinal vascular proliferation or tufts, called ridge formation, at stage 2, extraretinal fibrovascular proliferation from the ridge into the vitreous in stage 3, and traction retinal detachment at stage 4 and 5. Retinopathy of prematurity may spontaneously regress at stages 1 to 2. Rapidly deteriorating signs such as retinal arterial tortuosity and venous dilation in the posterior pole and vitreous haze are designated as plus disease and pre-plus disease, which can be used as a sign for aggressive retinopathy of prematurity. Retinal ablation, usually with laser photocoagulation, is applied to the avascular retina in the case that the ridge remains active. In the mid-1980s, the International Classification of Retinopathy of Prematurity was established [3-5], and avascular retinal ablation with photocoagulation or cryocautery at the recommended timing in retinopathy of prematurity was shown to be effective in leading to better visual outcome by the Early Treatment for Retinopathy of Prematurity trial [6,7].

In Japan, the effectiveness of xenon-arc photocoagulation and cryocautery in retinopathy of prematurity was reported on an empirical basis in 1968 by Nagata et al. [8-10] and in 1972 by Yamashita [11], respectively. In the mid-1970s, the Classification of Retinopathy of Prematurity by the Ministry of Health and Welfare in Japan, which was basically the same as the later International Classification, was established [12]. Xenon-arc photocoagulation and cryocautery became the standard of care in retinopathy of prematurity 10 years earlier compared with other countries [13,14]. We previously reported the up to 20-year visual outcome of 28 consecutive patients with retinopathy of prematurity who underwent xenon-arc photocoagulation and cryocautery in the 1970s [15]. In this study, we reported the up to 49 years visual outcome of 11 patients out of the 28 patients.

## Materials and methods

Retrospective review was made on the medical records of 11 consecutive patients who underwent xenon-arc photocoagulation for retinopathy of prematurity in the years 1974 to 1980 [14,15], and were followed up until the period from 2009 to 2025. Xenon-arc photocoagulation in all 11 patients was done by the second author and followed until the year 1997. Afterwards, these patients were followed and treated by the first author. There were no exclusion criteria in this study. This study was approved as a retrospective observational study by the Ethics Committee of Graduate School of Medicine, Dentistry, and Pharmaceutical Sciences, Okayama University, and Okayama University Hospital (No. 2507-056, July 18, 2025).

The International Classification of Retinopathy of Prematurity was used in this study [3-5]. The classification into zone I, II, and III retinopathy was omitted in this study because of the difficulty in differentiating zone I and II from our medical records [15], which used the classification system of Japan’s Ministry of Health and Welfare [12]. Xenon arc photocoagulation and cryocautery were usually done at stage 3, but were done at stage 2 when there were signs of plus disease [4,5]. Xenon-arc photocoagulation was done with the Model XC-5.50A (NIDEK Co. Ltd, Gamagori, Aichi, Japan) at a maximum power, in the size of 6 degrees, and in the exposure duration of 0.5 seconds. Transconjunctival cryocautery was done with a retinal probe (Amoils Cryo Unit, Keeler Instruments, Windsor, UK) at -70 to -50°C for 7-15 seconds. According to the standard method used at that time, scattered photocoagulations were placed on the ridge as well as on its avascular periphery [14,15]. In retinopathy of prematurity with plus disease, including type II and intermediate type, which were defined by the Classification of the Ministry of Health and Welfare [12] and corresponded to aggressive retinopathy of prematurity [4,5], overlapping photocoagulations were made on the ridge and to the avascular area. There was also a row of scattered photocoagulations in the area posterior to the ridge at stage 2, in all eyes with type II, and in some intermediate type eyes. The area of treatment was expressed as clock hours meridian, as used in the International Classification of Retinopathy of Prematurity [3-5].

Descriptions which were retrieved from the medical records were birth year, birthweight, gestational age at birth, corrected gestational age when photocoagulation was done, oxygen administration, stage, plus disease, clock hours of treatment with photocoagulation which were defined by the International Classification of Retinopathy of Prematurity, the additional treatment with cryocautery, the age at the last visit, best-corrected visual acuity and refractive errors at the last visit, and late complications. In the process of data extraction from medical records, a standardized form, which was basically the same as Table 1, was used for the data retrieval. The data, regarding the birth and treatment, were retrieved from the previous datasheet, which had been prepared in our previous study [15]. The retrieved data were checked once again on a different day by the first author in the validation step.

**Table 1:** Summary of 11 patients with retinopathy of prematurity who underwent xenon-arc photocoagulation in the year from 1974 to 1980. ICROP, International Classification of Retinopathy of Prematurity; NLP, no light perception; s, spherical; c, cylindrical; A, axis; A-ROP, aggressive retinopathy of prematurity.

Case no./sex	Birth year	Birth weight	Gestational age at birth	Corrected gestational age	Oxygen therapy	Eye	ICROP Stage (+: plus disease)	Clock hours of treatment with xenon-arc	Additional cryocautery	Age at last visit	Best-corrected visual acuity at last visit	Late complications
1/Female	1974	2300 g	36 weeks	48 weeks	No	Right	3	4	No	41 years	0.8 x s-1.0 c-0.75A180	No
						Left	3	4	No		1.2 x c-0.75A75	No
2/Female	1975	865 g	27 weeks	35 weeks	Yes	Right	3+	12	Yes, partial	47 years	1.0 x s-0.75 c-1.5A150	No
						Left	3+	12	Yes, partial		1.0 x s+1.5 c-2.5A30	No
3/Female	1975	1580 g	33 weeks	37 weeks	Yes	Right	3+	12	Yes, 360 degrees	46 years	1.2 x s-9.0 c-2.5A180	No
						Left	3+	12	Yes, 360 degrees		0.1 x s+4.0 c-4.0A180	Retinal detachment: vitrectomy, silicone oil tamponade and cataract surgery at 44 years
4/Male	1975	1050 g	30 weeks	36 weeks	Yes	Right	2+, A-ROP	12	No	49 years	0.5 x s-3.75 c-1.5A125	Cataract surgery at 36 years
						Left	2+, A-ROP	12	No		0.02 x s-1.5 c-1.0A50	Cataract surgery at 36 years
5/Male	1975	950 g	30 weeks	36 weeks	Yes	Right	1+, A-ROP	12	Yes, partial	49 years	NLP	Phthisis, Nystagmus
						Left	1+, A-ROP	12	Yes, 360 degrees		0.1 x s+10.0	Cataract surgery at 34 years. Vitrectomy: removal of in-the-bag intraocular lens dislocation at 47 years
6/Male	1975	1600 g	32 weeks	41 weeks	Yes	Right	3	3	No	43 years	1.5 x s-4.0 c-1.5A85	No
						Left	3	3	Yes, partial		1.5 x s-3.0 c-1.0A165	No
7/Male	1976	1005 g	27 weeks	35 weeks	Yes	Right	3+	12	Yes, 360 degrees	49 years	1.2 x s-3.75 c-1.0A180	Cataract surgery at 43 years
						Left	3+	12	Yes, partial		1.2 x s-5.25 c-1.0A180	Cataract surgery at 39 years. Vitreous hemorrhage with spontaneous absorption at 37 years
8/Male	1976	1600 g	32 weeks	37 weeks	Yes	Right	3+	12	Yes, partial	49 years	0.3 x s+5.5 c-4.0A180	Cataract surgery at 41 years. Retinal detachment: vitrectomy and silicone oil tamponade at 41 years
						Left	3+	12	Yes, partial		0.8 x s+0.5 c-1.0A90	Cataract surgery at 41 years
9/Female	1976	1390 g	35 weeks	41 weeks	Yes	Right	3	3	No	36 years	1.5 x s-8.0	No
						Left	2	No	No		1.5 x s-4.0	No
10/Female	1977	1350 g	30 weeks	53 weeks	Yes	Right	3	4	No	32 years	1.2 x s-3.25 c-3.0A180	Partial scleral buckling at 22 years
						Left	3	4	No		1.2 x c-1.5A20	No
11/Male	1980	1312 g	28 weeks	32 weeks	Yes	Right	2, A-ROP	12	No	44 years	0.8 x s-13.0 c-4.5A155	No
						Left	2, A-ROP	12	No		0.8 x s-12.0 c-2.5A25	Vitreous hemorrhage with spontaneous absorption at 42 years

## Results

**Figure 1:**
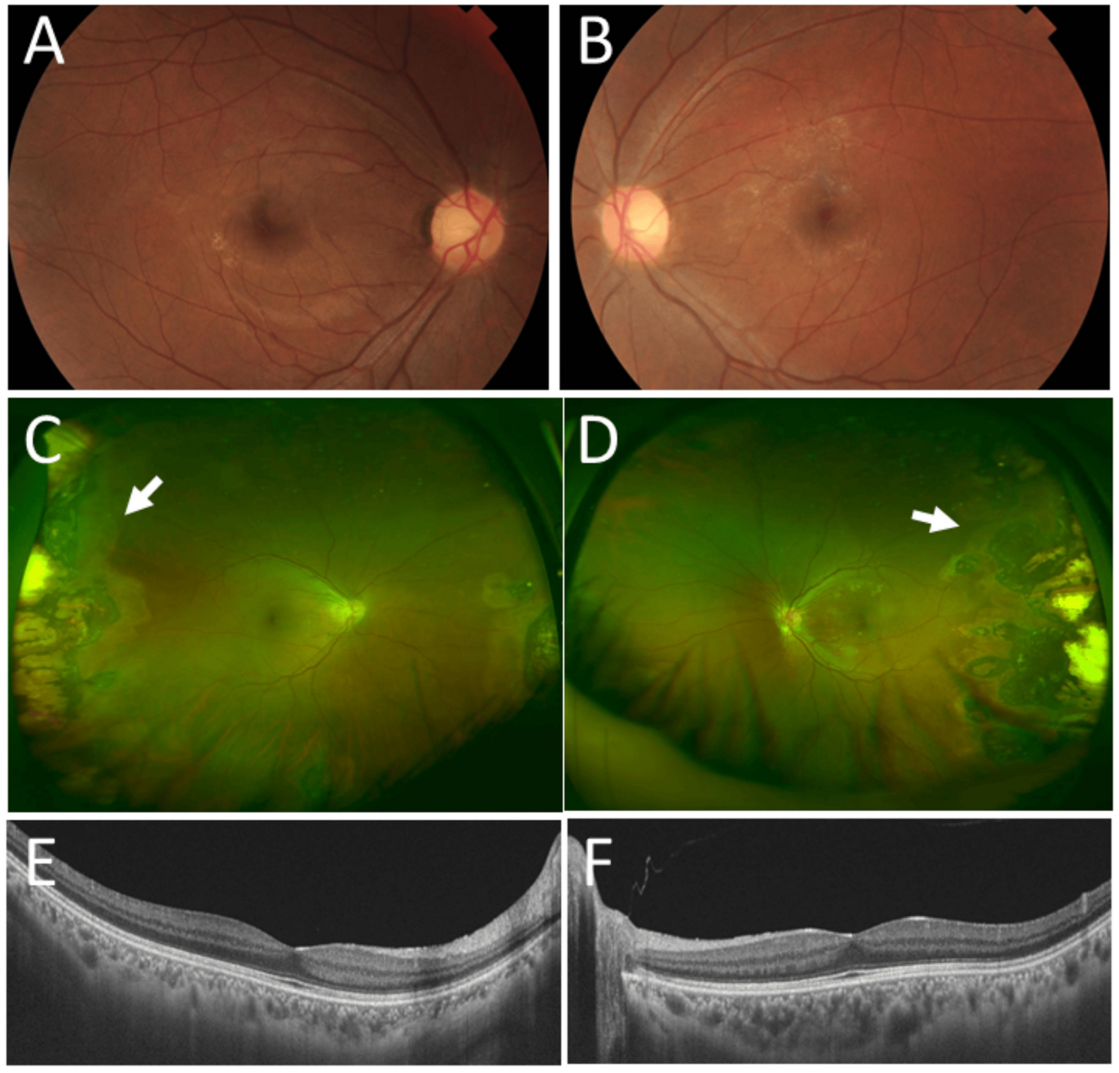
Clinical images in Case 1 and Case 2. Case 1. Fundus photographs at age 39 years, showing normal posterior pole in the right eye (A) and the left eye (B). Case 2. Wide-field fundus photographs (C, D) and horizontal section images of optical coherence tomography (E, F) at age 47 years, showing temporal peripheral photocoagulation scars (arrows) and normal macula in the right eye (C, E) and the left eye (D, F).

**Figure 2:**
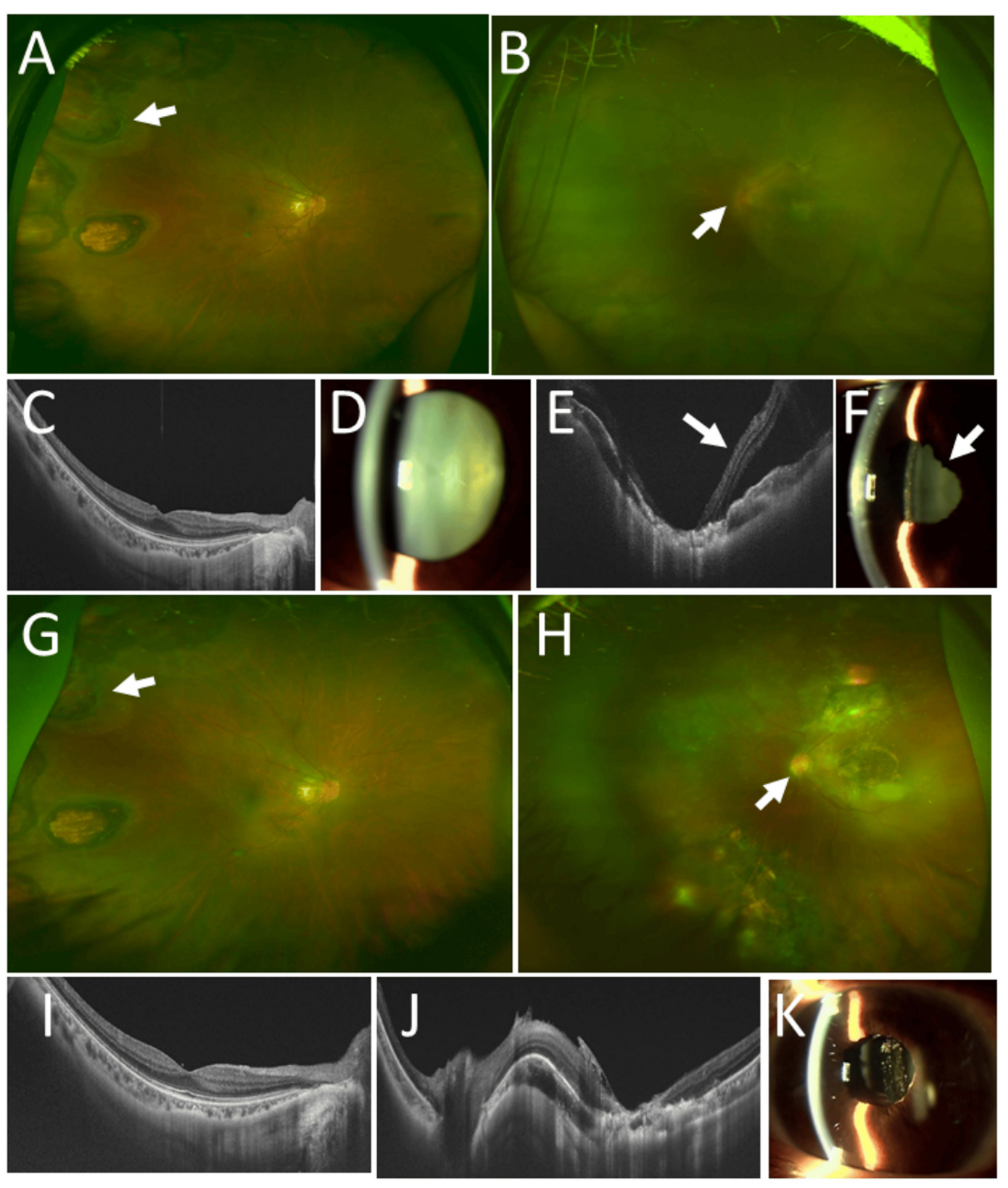
Clinical images in Case 3. Wide-field fundus photographs (A: right eye, B: left eye), horizontal section images of optical coherence tomography (C: right eye, E: left eye), slit-lamp photographs (D: right eye, F: left eye) at age 44 years. Note retinal detachment (arrows) except for the area with photocoagulation scars (B, E) and posterior iris synechia (arrow, F). Wide-field fundus photographs (G: right eye, H: left eye) and horizontal section images of optical coherence tomography (I: right eye, J: left eye) in both eyes, and slit-lamp photograph (K) in left eye 2 years later at age 46 years, showing no retinal detachment with silicone oil tamponade (J) and intraocular lens implantation (K). Arrows in A and G indicate photocoagulation scars in right eye. Arrow in H indicates optic disc in left eye.

**Figure 3:**
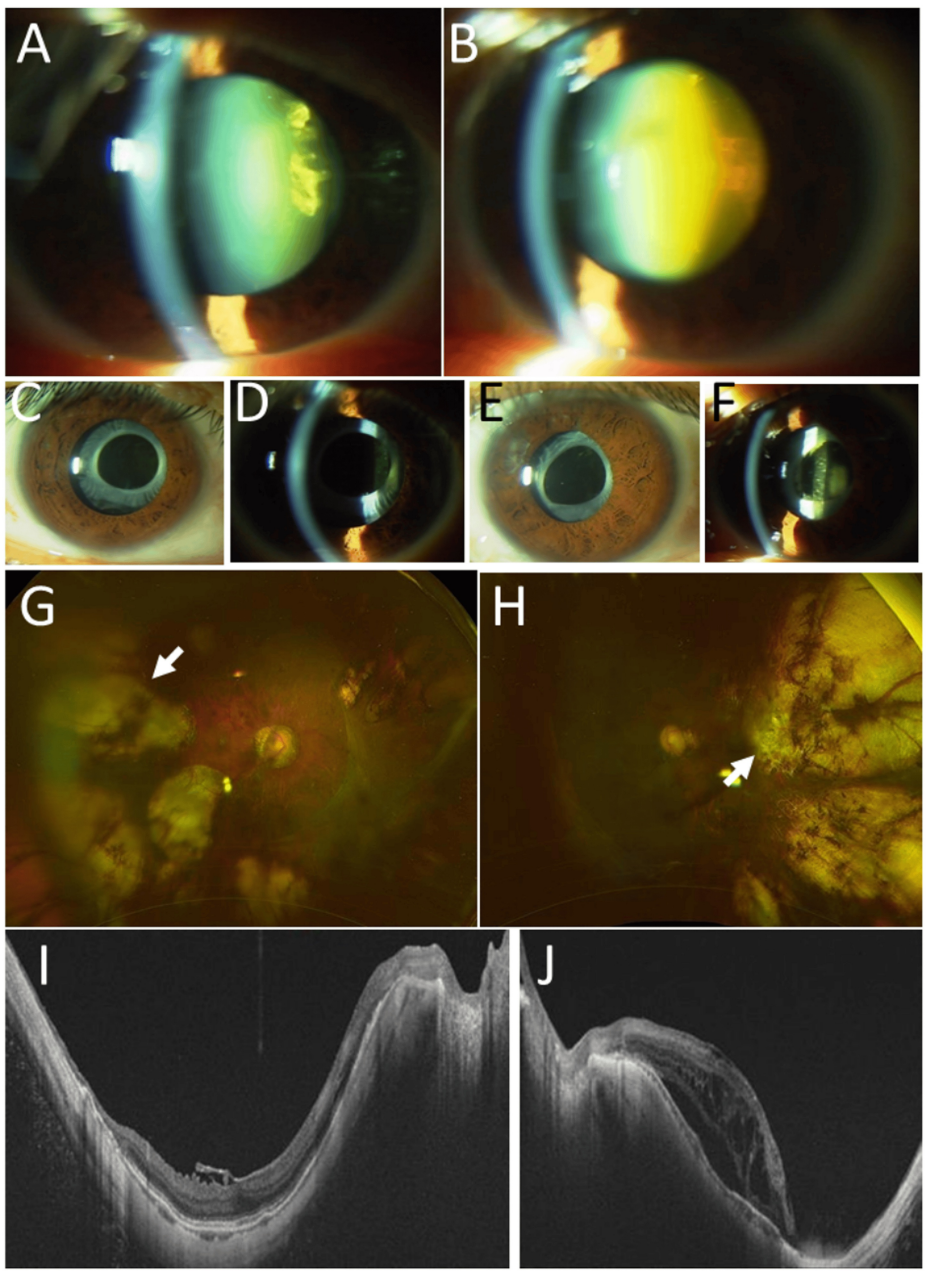
Clinical images in Case 4. Slit-lamp photographs, showing nuclear and posterior subcapsular cataract in right eye (A) and left eye (B) at age 36 years, intraocular lens implantation in right eye (C, D) and left eye (E, F) one year later at age 37 years. Wide-field fundus photographs (G: right eye, H: left eye) and horizontal section images of optical coherence tomography (I: right eye, J: left eye) at age 48 years, showing photocoagulation and cryocautery scars (arrows) near the macular area.

**Figure 4:**
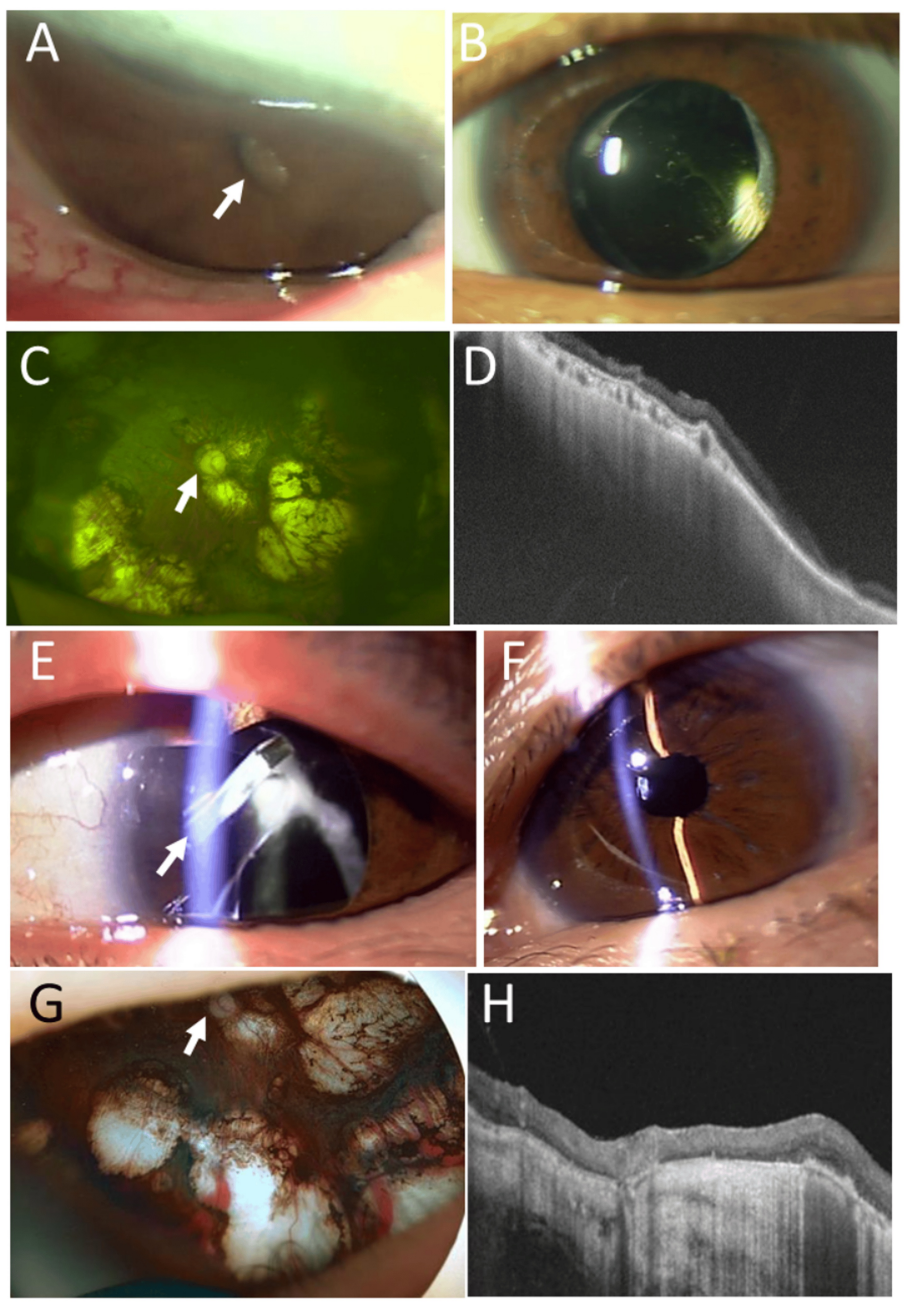
Clinical images in Case 5. Slit-lamp photographs in right eye (A) with pupillary occlusion (arrow) and intraocular lens implantation in left eye (B) at age 37 years. Wide-field fundus photograph (C) and horizontal section image of optical coherence tomography (D) in right eye at age 43 years, showing macular scars with pale-tone optic disc (arrow). Slit-lamp photograph (E) in left eye at age 47 years (E), showing dislocated intraocular lens in the capsular bag with lens haptic (arrow) visible under mydriasis. Slit-lamp photograph (F) at age 49 years, showing aphakia in left eye. Wide-field fundus photograph (G) and horizontal section image of optical coherence tomography (H) in left eye, showing macular scars with pale-tone optic disc (arrow).

**Figure 5:**
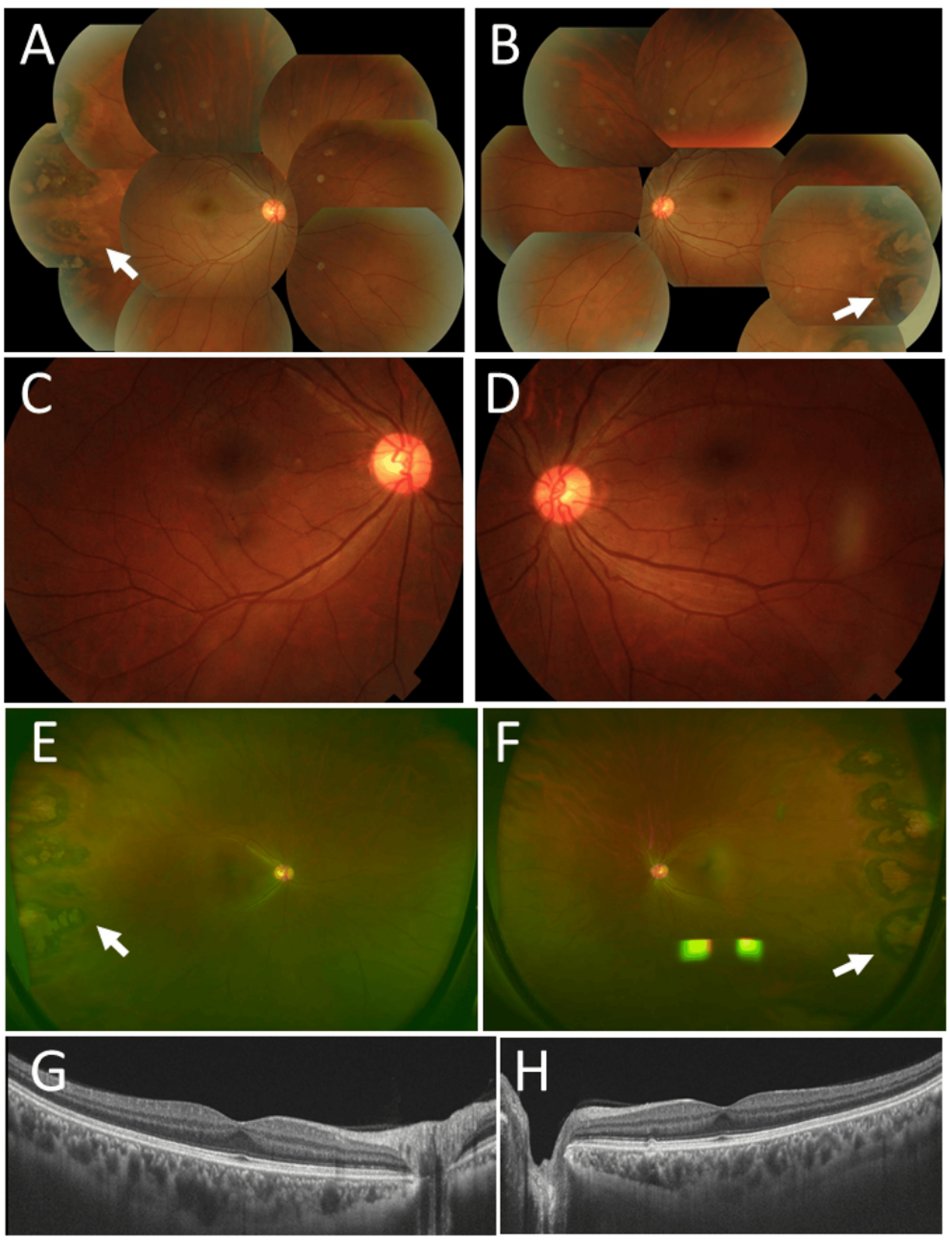
Clinical images in Case 6. Montaged fundus photographs in right eye (A) and left eye (B) at age 33 years, showing temporal peripheral photocoagulation scars (arrows) in both eyes. Fundus photographs in right eye (C) and left eye (D) at age 42 years, showing normal posterior poles. Wide-field fundus photographs and horizontal section images of optical coherence tomography in right eye (E, G) and left eye (F, H), showing temporal peripheral photocoagulation scars (arrows) and normal macular structure in both eyes.

**Figure 6:**
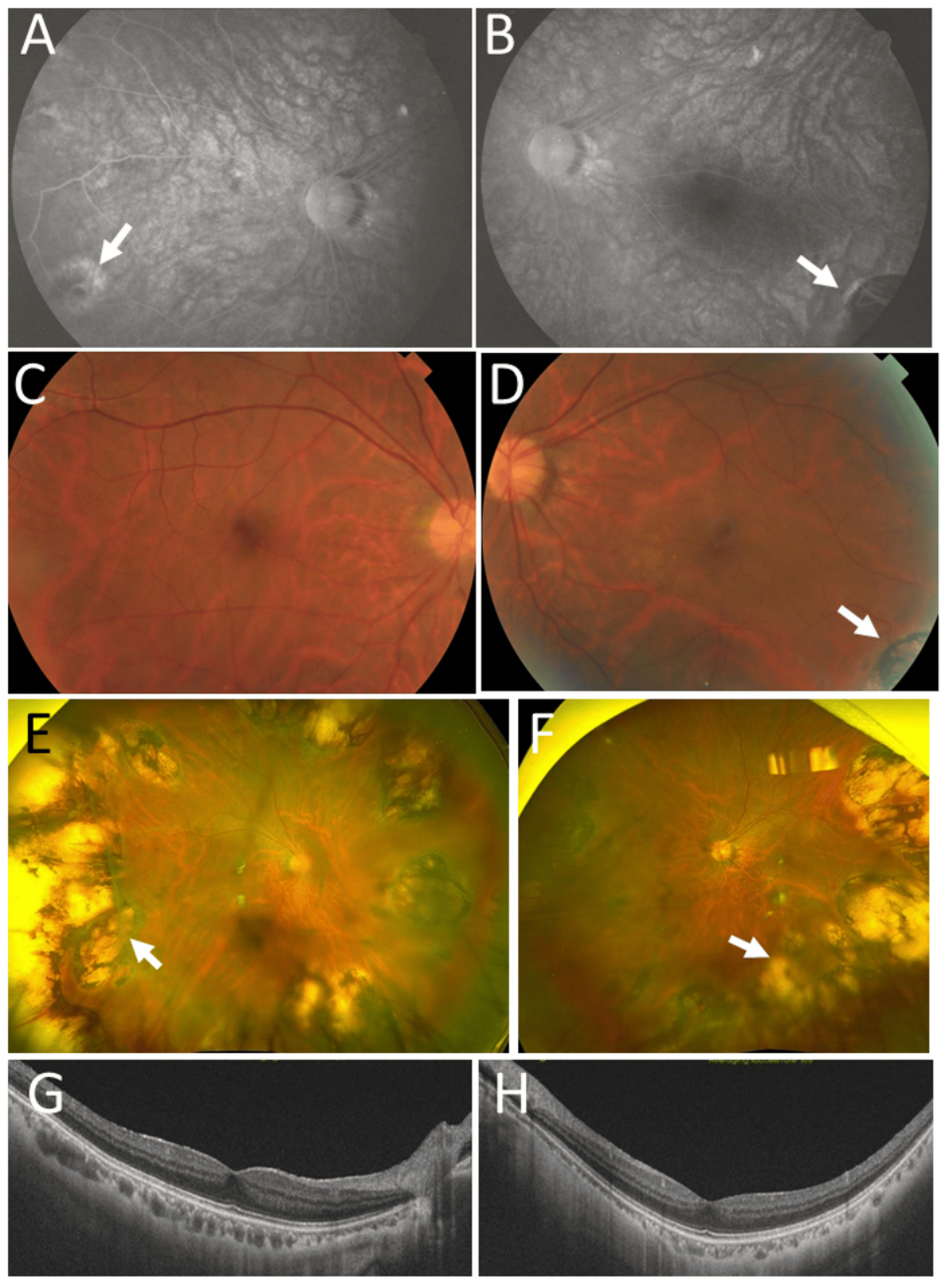
Clinical images in Case 7. Fluorescein angiograms on the late phase in right eye (A) and left eye (B) at age 18 years, showing photocoagulation scars temporal to the macular area in both eyes (arrows). Fundus photographs in right eye (C) and left eye (D) at age 37 years, showing normal posterior poles with a photocoagulation scar (arrow, D) in left eye. Wide-field fundus photographs and horizontal section images of optical coherence tomography in right eye (E, G) and left eye (F, H) at age 49 years, showing whole-circumference midperipheral photocoagulation scars (arrows) and normal macular structure in both eyes.

**Figure 7:**
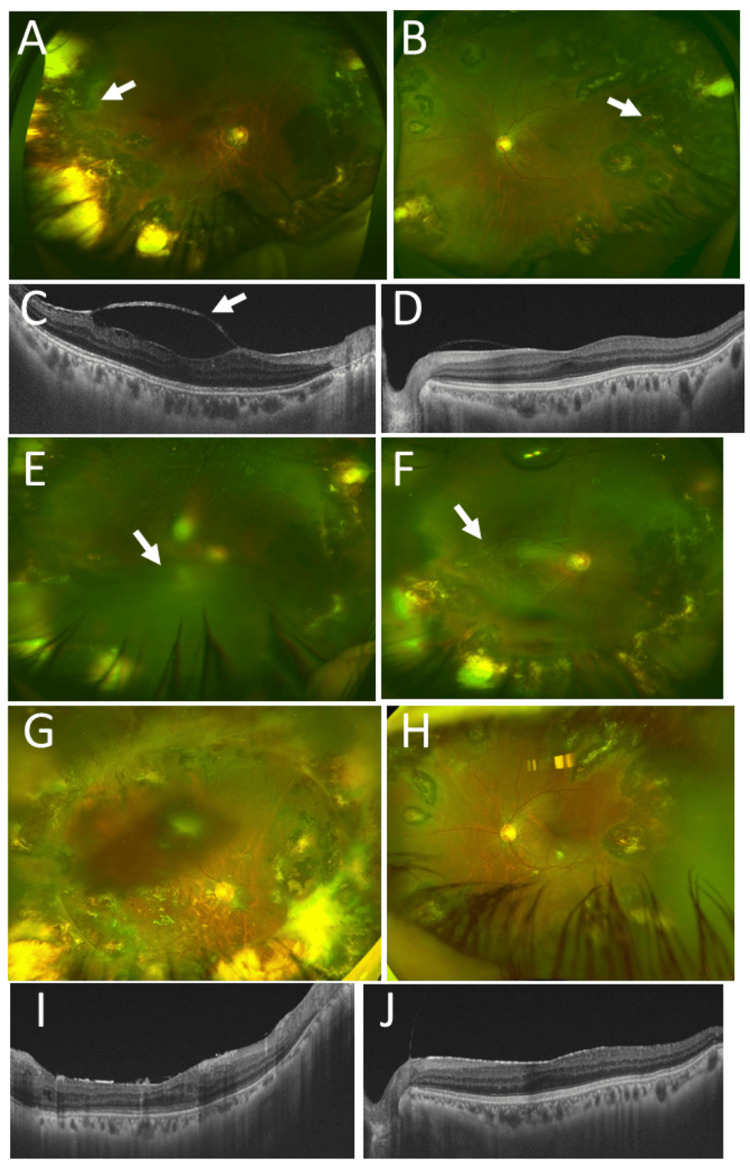
Clinical images in Case 8. Wide-field fundus photographs and horizontal section images of optical coherence tomography in right eye (A, C) and in left eye (B, D) at age 39 years, showing whole-circumference midperipheral photocoagulation scars (arrows) in both eyes with mild vitreomacular traction in right eye (arrow, C) in otherwise normal macular structure. Wide-field fundus photographs in right eye (E, F) at age 41 years, showing retinal detachment and the recurrence (arrows) one month after vitrectomy and gas tamponade. Wide-field fundus photographs and horizontal section images of optical coherence tomography in right eye (G, I) and left eye (H, J) at age 49 years, showing reattached retina (G) with no vitreoretinal traction (I) in right eye long after vitrectomy and silicone oil tamponade at age 41 years (F).

**Figure 8:**
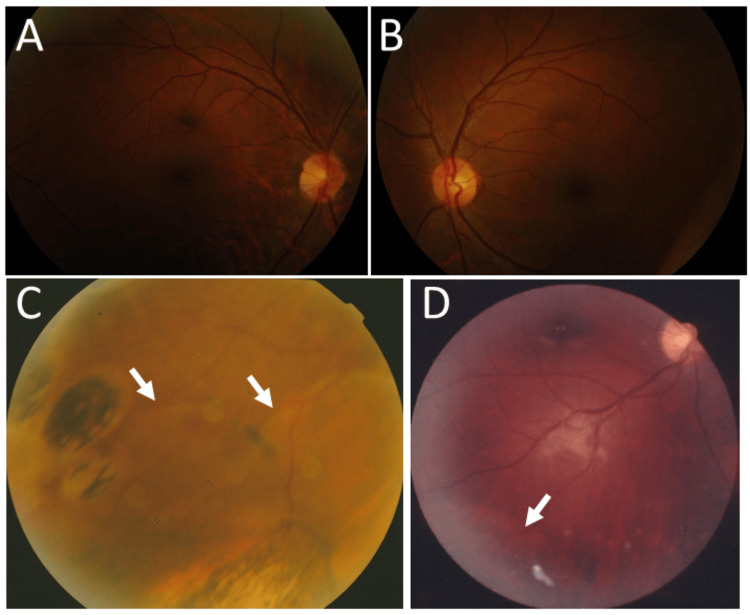
Clinical images in Case 9 and Case 10. Case 9. Fundus photographs in right eye (A) and left eye (B) at age 36 years, showing normal macula. Case 10. Fundus photographs in right eye at age 22 years, showing inferior temporal midperipheral retinal detachment (arrows, C) and residual subretinal fluid (arrow, D) along the slope of partial scleral buckling one month later.

**Figure 9:**
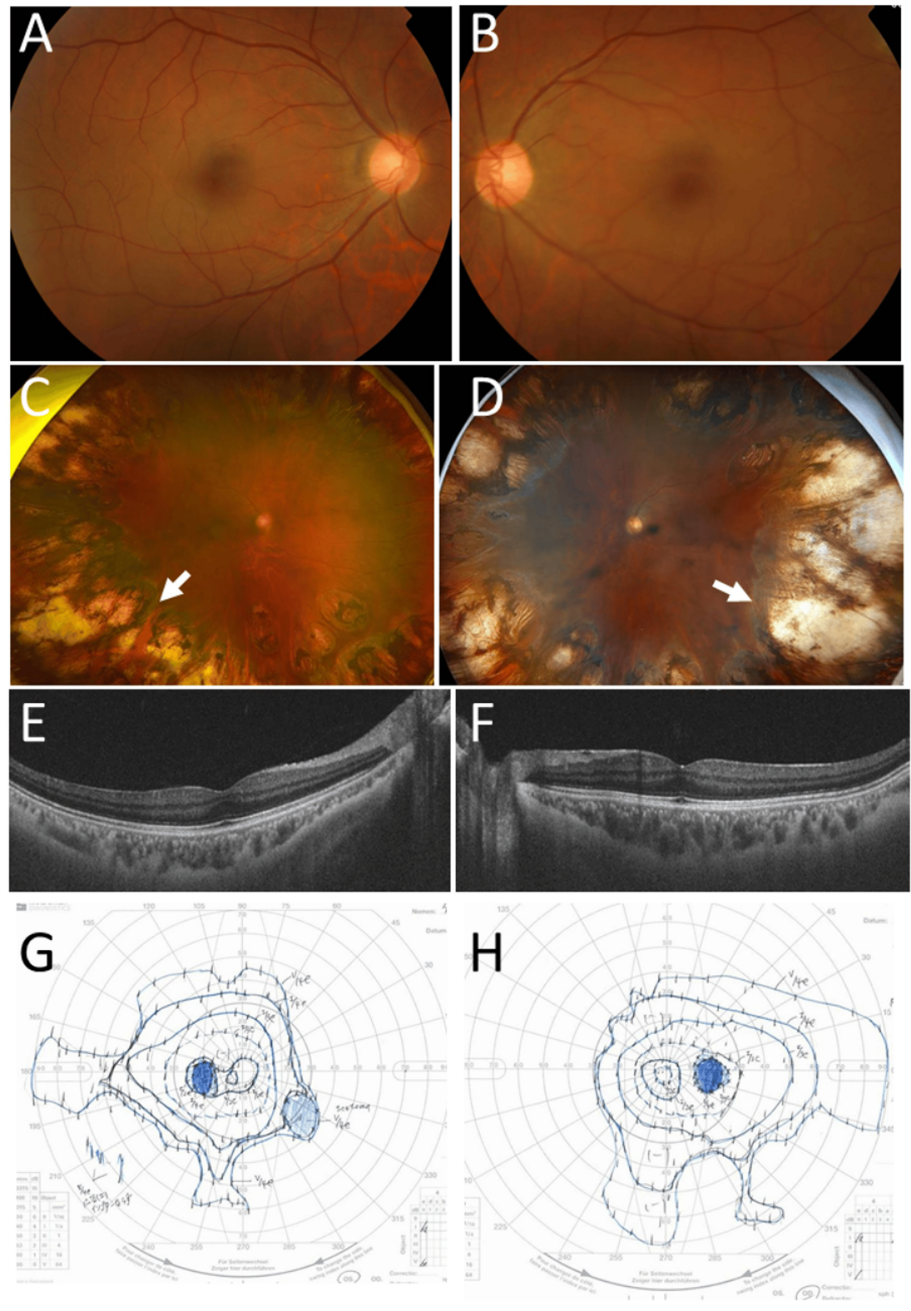
Clinical images in Case 11 Fundus photographs in right eye (A) and left eye (B) at age 28 years, showing normal posterior poles. Wide-field fundus photographs and horizontal section images of optical coherence tomography in right eye (C, E) and left eye (D, F) at age 44 years, showing whole-circumference midperipheral photocoagulation scars (arrows) and normal macular structure. Goldmann perimetry in left eye (G) and right eye (H) at age 43 years, showing indented visual fields caused by photocoagulation scars.

The clinical features in 11 patients are summarized in Table 1. Clinical images of these 11 patients in recent years are shown in Figures 1-9. Eight patients were born in the years 1975 and 1976, while each of the other three patients was born in 1974, 1977, and 1980, respectively. The birthweight ranged from 865 g to 2300 g at a median of 1350 g, and the gestational age at birth ranged from 27 weeks to 36 weeks at a median of 30 weeks. The corrected gestational age at the time of photocoagulation ranged from 32 weeks to 53 weeks, with a median of 37 weeks. Oxygen was given to all 11 patients, except for one who was born in the earliest year 1974.

Seven of the 11 patients showed stage 3 retinopathy of prematurity in both eyes, based on the International Classification of Retinopathy of Prematurity: four patients at stage 3 with plus disease had photocoagulation for 12 clock hours in both eyes, while the remaining three patients with no plus disease had photocoagulation in the limited area of 3 to 4 clock hours. In a pair of dizygotic twins born at 30 weeks of gestation, one twin (Case 5) with a birthweight of 950 g was at stage 1 with plus disease in both eyes, while the other (Case 4) with a birthweight of 1050 g was at stage 2 with plus disease in both eyes. Both twins underwent photocoagulation for 12 clock hours in both eyes since they were designated as aggressive retinopathy of prematurity. One patient (Case 11) showed stage 2 in both eyes with aggressive retinopathy of prematurity and had photocoagulation for 12 clock hours in both eyes. Another patient (Case 9) showed stage 3 in the right eye and stage 2 in the left eye and had photocoagulation for 3 clock hours only in the right eye. Additional cryocautery was applied in both eyes of the four patients at stage 3 with plus disease, in both eyes of one patient (Case 5) at stage 1 with plus disease, and in the left eye of another patient (Case 6).

The age at the last visit ranged from 32 to 49 years, with a median of 46 years. At the last visit, seven patients showed the best-corrected visual acuity in decimals of 0.8 or better with varying levels of myopic astigmatism. One (Case 5) of the dizygotic twins had no light perception in the phthisic right eye and the best-corrected visual acuity of 0.1 in the left eye with macular degeneration and nystagmus after he underwent cataract surgery with intraocular lens implantation at the age of 34 years and later had removal of the disclosed intraocular lens in the capsular bag at the age of 47 years. The other (Case 4) of the twin had the best-corrected visual acuity of 0.5 in the right eye and 0.02 in the left eye due to macular degeneration after he underwent cataract surgeries with intraocular lens implantation in both eyes at the age of 36 years. Two patients developed rhegmatogenous retinal detachment in one eye at the age of 44 years (Case 3) and 41 years (Case 8) and had vitrectomy with silicone oil tamponade, leading to poor levels of visual acuity of 0.1 and 0.3, respectively. Of these two patients, one patient (Case 8) developed retinal detachment in the right eye four months after uncomplicated cataract surgery with intraocular lens implantation. He initially underwent vitrectomy with gas tamponade, but one month later, he developed the recurrence of retinal detachment and this time underwent silicone oil tamponade. Based on this experience, the other patient (Case 3) underwent vitrectomy and silicone oil tamponade, combined with cataract surgery with intraocular lens implantation as an initial surgery.

Two patients (Case 7 and Case 11) experienced vitreous hemorrhage in one eye, which was absorbed spontaneously at the ages of 37 years and 42 years, respectively. One patient (Case 10) underwent partial scleral buckling for localized rhegmatogenous retinal detachment in the right eye. No patient used intraocular pressure-lowering eyedrops.

## Discussion

In our previous study, which was published in 1997, 52 eyes of 28 patients who underwent xenon-arc photocoagulation in addition to or without cryocautery were classified into two groups from the viewpoint of final best-corrected visual acuity up to 20 years later: 43 eyes showed the visual acuity of 0.6 or better, while nine eyes showed the visual acuity of 0.2 or worse [15]. Poor visual outcome resulted from macular degeneration, which occurred either as an isolated small focus extending circumferentially around the fovea or as a result of extension from the temporal degeneration caused by the treatment. Poor visual outcome was significantly associated with low birthweight, the presence of plus disease, treatment of more clock hours of the fundus, treatment inside the vascular arcade, and treatment with 360-degree cryocautery.

The purpose of the present study was to reveal late complications of retinopathy of prematurity with xenon-arc photocoagulation in up to 49 years of follow-up. Our earliest experience on retinopathy of prematurity in the 1970s was, of course, different from the later and recent trend of smaller birthweight and earlier birth at the gestational age [16]. Furthermore, tissue oxygen is now monitored, and oxygen administration is avoided or well-controlled. Of the 11 patients involved in this study, four patients showed no complication and maintained normal levels of visual acuity in both eyes. Four patients in their 30s or 40s underwent cataract surgery in both eyes, except for one patient (Case 5), only in one eye, since his other eye was phthisic. Three patients developed retinal detachment in the unilateral eye. Two patients developed vitreous hemorrhage without retinal detachment, which absorbed spontaneously. These complications, cataract, retinal detachment, and vitreous hemorrhage, have also been described in other studies [17-20].

Retinal detachment is a major late complication that has to be managed on an individual basis [21-25]. Two patients (Case 3 and Case 8) underwent vitrectomy with silicone oil tamponade for extensive retinal detachment involving the posterior pole. In the first patient (Case 8) at the age of 41 years, vitrectomy with gas tamponade resulted in retinal detachment again in one month, and silicone oil tamponade was performed to attain long-term retinal attachment. He had the visual acuity of 1.0 in the right eye around the age of 20 years in our previous study [15] and maintained the visual acuity of 0.3 in the operated-on right eye with silicone oil tamponade for 8 years. In the second patient (Case 3) at the age of 44 years, vitrectomy with silicone oil tamponade was chosen as an initial surgery [26], based on the experience in the first patient. She had the visual acuity of 0.1 in the left eye due to macular degeneration around the age of 20 years in our previous study [15] and gained the same level of visual acuity in the operated-on left eye. Silicone oil was not removed in these two patients due mainly to the years of the COVID-19 pandemic. Additionally, silicone oil was stable with no emulsification, and the patients were satisfied with their vision.

A major limitation in this study is the inclusion criteria. The cohort of 28 patients with retinopathy of prematurity who underwent xenon-arc photocoagulation in our previous study [15] decreased to 11 patients in this study, who were followed up in the same institution for a long-term period. A large part of the patients in our previous study moved to other cities, usually in their late teens or twenties, for higher education, work, or marriage, and were lost to follow-up. Naturally, this study cannot specify the incidence of cataract, retinal detachment, and vitreous hemorrhage as late complications of retinopathy of prematurity with xenon-arc photocoagulation with or without additional cryocautery.

Another limitation in this study is the method of analysis, where original descriptions in the classification system by the Japanese Ministry of Health and Welfare were converted to the International Classification system. In this conversion, the zone classification was omitted since it was difficult to accurately define the zone based on the old medical records [15]. Under the circumstances, the main strength of this study could be the exceptionally long follow-up period, which would provide a unique longitudinal perspective with clinical images as a valuable asset [27,28]. However, the study might be significantly hampered by its retrospective and descriptive nature and also by a limited sample size. The retrospective design would be vulnerable to missing data, and the unexplored impact of confounding variables like ocular surgeries might be present in this study.

## Conclusions

Photocoagulation or cryocautery, or their combinations, are the standard of care for retinopathy of prematurity at the preferred and recommended timing, which is based on the International Classification of Retinopathy of Prematurity. In Japan, the effectiveness of xenon-arc photocoagulation and cryocautery in retinopathy of prematurity was reported on an empirical basis first in 1968, and became the standard of care in retinopathy of prematurity in the 1970s, 10 years earlier when compared with other countries. In this study, we reported the up to 49 years visual outcome of 11 patients with retinopathy of prematurity who underwent xenon-arc photocoagulation and cryocautery in the 1970s. Most of the patients with xenon-arc photocoagulation for retinopathy of prematurity in the 1970s maintained standard levels of visual acuity up to 49 years in follow-up. Cataract, retinal detachment, and vitreous hemorrhage were noted as late complications and were coped with on an individual basis.
